# Blood‐based proteomic profiling reveals context‐dependent changes in BCL2‐associated signaling during taxane therapy in breast cancer patients

**DOI:** 10.1002/2211-5463.70239

**Published:** 2026-03-26

**Authors:** Saira Munshani, Eiman Y. Ibrahim, Rozalyn L. Rodwin, Leah M. Ferrucci, Kim Blenman, Maryam Lustberg, Barbara E. Ehrlich

**Affiliations:** ^1^ Department of Pharmacology Yale School of Medicine New Haven CT USA; ^2^ Department of Pediatrics (Hematology/Oncology) Yale School of Medicine New Haven CT USA; ^3^ Department of Chronic Disease Epidemiology and Yale Cancer Center Yale School of Public Health New Haven CT USA; ^4^ Department of Medicine (Medical Oncology) Yale School of Medicine New Haven CT USA; ^5^ Department of Computer Science, School of Engineering and Applied Science Yale University New Haven CT USA; ^6^ Present address: Department of Endocrinology University of Missouri Columbia MO USA

**Keywords:** apoptosis, breast cancer, calcium signaling, paclitaxel, senescence

## Abstract

The quality of life for many cancer survivors is compromised due to severe, long‐lasting side effects of chemotherapy. As part of a pilot, prospective, non‐interventional study to examine the side effects of chemotherapy in breast cancer patients, we examined the change in protein expression in blood collected from patients before and after treatment with taxanes for 12 weeks. Protein expression was measured with reverse phase proteomic arrays (RPPA), which revealed divergent changes in apoptosis, senescence, and calcium signaling‐related proteins depending on treatment setting (neoadjuvant vs. adjuvant). The largest change identified was BCL2 (B‐cell lymphoma 2), a founding member of the BCL2 family of proteins that regulate apoptosis. Other proteins regulated by BCL2, including RB1 (retinoblastoma protein 1) and NLRP3 (NLR family pyrin domain containing 3) changed significantly over the course of treatment. These differences are consistent with intracellular calcium signaling dysregulation and activation of stress‐response pathways that overlap with senescent‐associated secretory phenotype (SASP)‐like signaling, which has been implicated in cancer recurrence. To contextualize these observations, we generated Kaplan–Meier survival curves using publicly available proteomics data from The Cancer Proteome Atlas (TCPA). This work aims to demonstrate how blood‐based proteomics can serve as a non‐invasive method to monitor systemic physiological shifts during cancer therapy, offering a framework for generating hypotheses about chemotherapy timing and long‐term outcomes.

## Graphical abstract

Chemotherapy side effects significantly impact cancer survivors' quality of life. Using protein levels in blood samples from breast cancer patients before and after 12 weeks of taxane treatment, we detected treatment‐dependent changes in calcium signaling and aging pathways associated with cancer recurrence. These findings support blood‐based proteomics as a non‐invasive framework for monitoring systemic responses and informing long‐term outcomes.

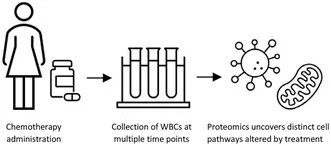

**Abbreviations**
BCL2: B‐cell lymphoma 2
DDR: DNA damage response
ER: endoplasmic reticulum
GRP75: glucose‐related protein 75
IL6: interleukin‐6
ITPR: inositol tris‐phosphate receptor
MAM: mitochondrial‐associated ER membrane
NLRP3: NLR family pyrin domain containing 3
NRF2: nuclear factor erythroid 2‐related factor 2
OS: overall survival
QC: quality criteria
RB1: retinoblastoma protein 1
ROS: reactive oxygen species
RPPA: reverse phase proteomic arrays
SASP: senescent‐associated secretory phenotype
TCGA: The Cancer Genome Atlas
TCPA: The Cancer Proteome Atlas
VDAC: voltage‐dependent anion channel

## Introduction

Taxanes are a family of antineoplastic agents that include paclitaxel and docetaxel, commonly used as chemotherapy in several cancer types, including breast, ovarian, and lung [1]. Along with reducing the tumor size, administration of these drugs leads to changes in the expression of a number of proteins [2, 3, 4, 5]. One of the proteins impacted by paclitaxel in breast cancer cells is BCL2, an anti‐apoptotic agent that regulates calcium release from the intracellular calcium stores in the endoplasmic reticulum (ER) [6]. BCL2 prevents apoptosis through a complex network that includes the inhibition of RB1 (retinoblastoma protein 1), NLRP3 (NLR family pyrin domain containing 3), and the activation of GRP75 (glucose‐related protein 75; also known as HSPA9) [7]. RB1 functions to promote cellular senescence, and its phosphorylation state is necessary for determining a cell's fate [8]. NLRP3 is a protein involved in the inflammasome, and its activation leads to cell death [9]. GRP75 is a protein that facilitates contact between the ER and mitochondria [10], allowing preferential movement of calcium between these organelles.

Calcium signaling dysregulation leads to adverse side effects during taxane‐based chemotherapy [11, 12, 13]. In general, calcium signaling is a fundamental process that plays a critical role in cell growth, survival, and death. Alterations in calcium signaling alter many physiological processes which lead to diseases including heart failure, cancer, and neurodegenerative diseases [11, 14, 15]. Relevant to cancer therapy, calcium signaling dysregulation can engage cell stress programs that, depending on context, may include senescence‐associated secretory phenotypes (e.g., SASP signaling) or apoptosis [16]. The loss of calcium homeostasis is a stress that induces senescence, a state that is the consequence of the changes in the complex interactions between ER and mitochondrial functions [17]. These calcium and stress‐induced changes can lead to negative side effects from chemotherapy [18, 19]. Adverse responses include peripheral neuropathy and cognitive impairment [11, 12] and a senescent state where cancer cells become resistant to chemotherapy and radiation [13]. Mitigating the adverse effects of chemotherapy, including neuropathy, senescence, and cognitive impairment, is necessary to improve patients' overall health and to avoid treatment adjustments that can impact survival [20]. The aim of this pilot study was not statistical inference, but to evaluate whether longitudinal blood‐based proteomics could detect treatment‐associated shifts in stress‐response signaling within individual patients.

In this short report, we examine differences in protein expression of proteins related to apoptosis, senescence, and calcium signaling using samples obtained during a small prospective clinical study that monitored the development of chemotherapy‐induced neuropathy in patients with breast cancer who received adjuvant and neoadjuvant taxane‐based chemotherapy [21]. Neoadjuvant chemotherapy is a commonly used option for breast cancer patients, and it is defined as pharmacological treatment administration prior to surgical intervention [22]. Adjuvant chemotherapies are designed to be delivered after the primary treatment, often surgery, to help to eliminate malignant cells to reduce the chances of recurrence [23]. The largest differences between adjuvant and neoadjuvant groups occurred with proteins related to the pro‐apoptotic protein BCL2 and to intracellular calcium signaling. The number of subjects in the prospective study was small, but the results are compatible with conclusions from analysis of large‐scale The Cancer Proteome Atlas (TCPA) data. We use the BCL2 signaling network as an example of how non‐invasive proteomics can inform hypotheses about the systemic cell stress responses in cancer patients. This analysis does not lead to definitive conclusions about causality or mechanism. Rather, we pose proteomics as a tool that can be used to generate hypotheses and aid future treatment decisions. Our goal is to illustrate the utility of non‐invasive profiling of blood cells as a tool for capturing the dynamic physiological changes which occur during chemotherapy administration.

## Methods

We carried out a prospective clinical study of peripheral neuropathy and cognitive function in taxane‐treated breast cancer patients with methods previously described [21]. Studies were approved by the Yale Cancer Center Human Research Ethics Committee (NCT03872141), and written informed consent was obtained in accordance with the Declaration of Helsinki. Patients were recruited from 08 Aug 2017 to 11 Mar 2020. All data were de‐identified. Blood was collected in heparinized tubes before the first paclitaxel/docetaxel treatment (baseline) and again at 12 weeks during treatment. White blood cells were isolated, frozen, and stored at −80 °C.

To supplement the peripheral neuropathy and cognitive tests reported previously [21], white blood cell lysates were sent to the MD Anderson Functional Proteomics RPPA Core in March 2021. Processing followed the Core's standard pipeline. In brief, lysates were denatured, adjusted to a uniform concentration, serially diluted to maintain a linear detection range, and printed in technical replicates on nitrocellulose slides. Slides included designated control lysates for batch adjustment and a 32–cell line “mixed lysate” dilution series for spatial correction and curve fitting. Arrays were probed with monospecific, pre‐validated antibodies; only antibodies meeting Core quality criteria (QC score ≥ 0.8) were advanced to analysis. Signal was captured with tyramide amplification/DAB, background‐corrected, and fit to a supercurve. Values were linearly normalized to internal controls by the Core. We did not apply any additional post‐Core normalization or transformation.

Of the 13 enrolled participants, 7 contributed paired Week 0 and Week 12 samples suitable for RPPA. The remaining cases lacked a usable paired time‐point due to missed collection windows, insufficient specimen volume, or Core QC failure (e.g., suboptimal staining/array performance). Primary analyses were restricted to within‐patient paired comparisons to minimize bias from incomplete longitudinal sampling. In addition, baseline‐only samples were not pooled with paired data.

Week 0 refers to the pre‐chemotherapy baseline prior to the first paclitaxel/docetaxel dose. For neoadjuvant patients, Week 0 was pre‐surgery. For adjuvant patients, Week 0 was obtained approximately 10 weeks after definitive surgery (partial mastectomy or lumpectomy) and prior to initiating chemotherapy. Because adjuvant baselines were post‐operative, protein levels—including BCL2 in circulating WBCs—may reflect post‐operative systemic physiology rather than a true pre‐treatment state. Accordingly, we present neoadjuvant (*n* = 5) and adjuvant (*n* = 2) subgroups separately and interpret cross‐group differences with caution. A complete list of profiled proteins is provided in our prior report [21].

The Cancer Genome Atlas (TCGA) RPPA BRCA Invasive Carcinoma dataset (downloaded January 2023) was used to evaluate associations between protein expression and survival at scale [24, 25, 26]. TCGA RPPA does not specify neoadjuvant vs adjuvant treatment status; results are therefore interpreted as cohort‐level associations. TCPA data and KMplotter cutoffs were used to generate Kaplan–Meier plots for proteins selected based on the prospective study. All datasets used were either publicly available or are included in [21].

### Limitations

We were limited by the size of the cohort from the prospective study, which was seven patients. Thus, the patient data and the conclusions are more reminiscent of a case series as opposed to a definitive, large‐scale cohort study. Because this was a prospective pilot study with predefined enrollment and biospecimen availability, expansion to additional patients or other taxane‐sensitive tumor types was not feasible within the scope of this work. Although the breast cancer cohort included diverse receptor subtypes, this heterogeneity reflects real‐world clinical populations and underscores the systemic nature of the stress‐response signaling detected in circulating white blood cells. As analyses were performed on circulating white blood cells rather than tumor tissue, observed proteomic changes likely reflect systemic host responses to chemotherapy rather than tumor‐intrinsic subtype‐specific signaling.

In addition, the number of validated proteins included in the RPPA Core is limited to 484 proteins, primarily proteins related to cancer initiation and progression. Therefore, not all the proteins involved in the pathways related to the BCL2 pathway were available for analysis. In addition, although paclitaxel and docetaxel differ in pharmacokinetics and toxicity profiles, both are microtubule‐stabilizing taxanes with overlapping mechanisms of action; given the small cohort size, we treated taxane exposure as a shared class effect and interpret results cautiously. We also did not have access to pre‐surgical baselines of the adjuvant‐treated patients and recognize that partial mastectomies or lumpectomies can cause an increase in BCL2. Because of these limitations, we chose not to perform statistical comparisons. Rather, we frame the observed divergences in protein expression as preliminary and hypothesis‐generating. Finally, because WBCs are a mixed population and RPPA captures bulk protein abundance, shifts may reflect changes in cell composition and/or activation state rather than within‐cell fate changes.

## Results

The patients from the prospective clinical study were divided into two groups: neoadjuvant chemotherapy (*n* = 5) and adjuvant chemotherapy (*n* = 2). The relative change in protein level was calculated from baseline (0 weeks) to end of treatment (12 weeks). Note that baseline levels for neoadjuvant‐treated patients do not include any pre‐treatment data or other metrics prior to the 12‐week course of taxane treatment [21]. The expression of the following proteins is highlighted because there was an opposite trend between the neoadjuvant and adjuvant‐treated patients. The adjuvant‐treated patients did not receive any chemotherapy prior to the 12 weeks of paclitaxel/docetaxel; instead, they just received either a partial mastectomy or lumpectomy. It should be noted that the adjuvant‐treated group did receive a slightly increased dosage of paclitaxel/docetaxel during the 12 weeks, but the treatment regimen for all patients during the 12 weeks was limited to combinations of DDAC and trastuzumab with paclitaxel/docetaxel (Table 1). Additional demographic data are shown in Table S1.

**Table 1:** Patient‐specific details on HER2/ER/PR status, chemotherapy agents used, weekly dosage of taxol, previous therapies, and weeks post‐op (see Table S3 for demographics).

Patient	HER2/PR/ER status	Chemotherapy agents	Taxol dosage	Previous therapy	Weeks post‐op
*CIPN06*	Triple‐positive	Taxol 80 × 12 then DDAC	Not in chart		
*CIPN08*	Triple‐negative	Taxol 80 × 12 then DDAC	138 mg in 0.9% NaCl		
*CIPN09*	HER2+, PR−, ER−	DDAC then Taxol ×12	Not in chart		
*CIPN10*	HER2−, PR+, ER+	Taxol 80 × 12 then DDAC	138 mg in 0.9% NaCl		
*CIPN11*	HER2−, PR+, ER+	DDAC then Taxol ×12	156 mg in 0.9% NaCl	Lumpectomy	13 weeks, 5 days
*CIPN12*	Triple‐positive	DDAC then Taxol ×12	180 mg in 0.9% NaCl	Partial mastectomy	9 weeks, 3 days
*CIPN13*	HER2−, PR+, ER+	Taxol 80 × 12 then DDAC	138 mg in 0.9% NaCl		

### Main focus: BCL2's relationship with NLRP3, GRP75, and RB1

**Fig. 1:**
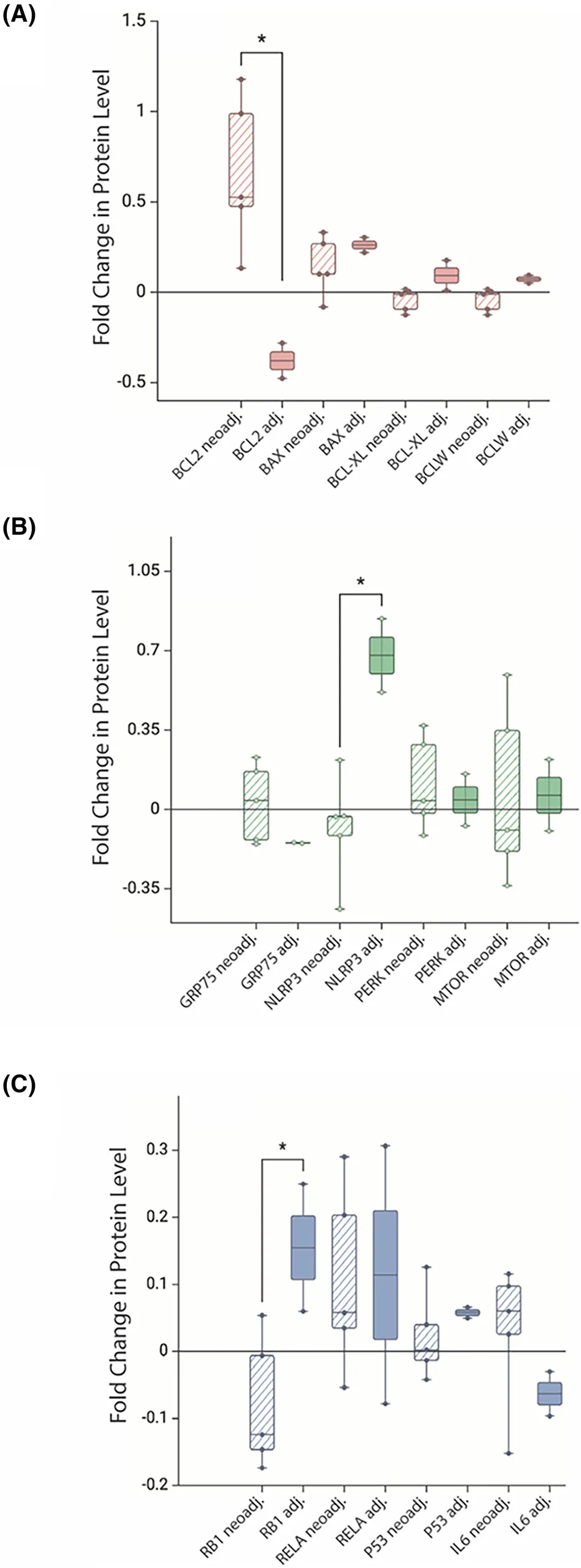
(A–C) Relative change in protein expression for neoadjuvant (*n* = 5) and adjuvant (*n* = 2) patients over the course of 12 weeks of treatment. (A–C) demonstrate proteins with notable changes within the BCL2 family (A), calcium signaling‐associated proteins (B), and senescence‐associated proteins (C). For proteins with a significant difference (see Table S3 for descriptive statistics) in expression after treatment between neoadjuvant and adjuvant groups. Data are presented as mean ± SD. Statistical comparisons were made using a paired *t*‐test; asterisks denote *P* < 0.05.

After dividing the patients into adjuvant and neoadjuvant treatment groups, data were sorted to identify proteins with a greater than statistically significant difference in the relative change in protein level between the two treatment groups. Of the 484 proteins evaluated by RPPA, the protein with the largest difference between neoadjuvant‐ and adjuvant‐treated patients was BCL2 (Fig. 1A). A list of the proteins with the largest differences shows a bias toward proteins in the BCL2 family, calcium signaling complex (Fig. 1B), and senescence pathway (Fig. 1C). Of the top hits, neoadjuvant‐ and adjuvant‐treated patients showed a distinct and opposite trend in protein expression for BCL2, NLRP3, and RB1. For adjuvant‐treated patients, BCL2 decreased by 37% (q1: −47%, q3: −28%), NLRP3 increased by 68% (q1: 51%, q3: 84%), and RB1 increased by 15% (q1: 6%, q3: 21%). For neoadjuvant‐treated patients, BCL2 increased by 67% (q1: 39%, q3: 103%), NLRP3 decreased by 8% (q1: −19%, q3: 3%), and RB1 decreased by 12% (q1: 0%, q3: −15%) (Tables S2, S3). These directional differences may reflect variability in pre‐ and post‐ surgery physiological states, leading to the induction of distinct cellular programs. Although these results reached statistical significance, the small sample size warrants caution. As mentioned earlier, these observations are primarily valuable to generate hypotheses based on published evidence. Previous reports indicate that paclitaxel treatment of cancer cell cultures lead to phosphorylation of BCL2 [27], a modification that determines a cell's fate [8]. Furthermore, BCL2‐mediated suppression of apoptosis can permit RB1‐dependent cell cycle arrest or senescence programs. RB1 functions as a central integrator of cell cycle arrest and apoptotic decisions, and its activity influences cellular reliance on BCL2‐family–mediated survival signaling [8, 28, 29].

### The BCL2 family

Given that adjuvant and neoadjuvant patients showed the greatest divergence in BCL2 protein level (Fig. 1A), we analyzed the available BCL2 family members in the patient RPPA data. BAX, BCL‐XL, BCLW, and BIM showed small differences between the two cohorts (Fig. 1A). For all these members of the BCL2 family, except BIM and PUMA (not included), the neoadjuvant patients had a slight decrease in protein levels relative to the adjuvant patients (Table S2).

Prior cell line studies of taxane response frequently emphasize paclitaxel‐induced phosphorylation of BCL2, rather than changes in total protein abundance, as a determinant of cell fate [27]. Consistent with this, reports of total BCL2 expression following taxane exposure are heterogeneous and context‐dependent across experimental models [6]. Our RPPA platform quantifies total protein levels in circulating white blood cells and therefore complements, but does not directly replicate, mechanistic tumor cell line studies that focus on post‐translational regulation [26]. Sustained BCL2‐family signaling has also been implicated in taxane resistance and therapy‐associated senescence in preclinical systems [30].

BCL2 is central to anti‐apoptotic processes through its ability to interfere with the actions of various protein partners: BCL‐XL, BAX, BCLW, and BIM. BCL‐XL is directly involved with mitochondrial homeostasis and has been reported to prevent apoptosis and control breast cancer cell migration via mitochondrial reactive oxygen species (ROS) formation [31]. Furthermore, it has been shown that nuclear factor erythroid 2‐related factor 2 (Nrf2) binds to the BCL‐2 gene antioxidant response element to control both BCL2 activity and cellular apoptosis [32] and also promotes chemoresistance [33]. In general, adjuvant patients had an increase in the expression levels of these pro‐apoptotic proteins, whereas neoadjuvant patients had a decrease in expression levels, suggesting that pro‐apoptotic behavior is downregulated when BCL2 levels increase. BCL2 also impacts the behavior of the inositol tris‐phosphate receptor (ITPR), where BCL2 binds to and inhibits the calcium channel activity of the intracellular calcium channel formed by ITPR [34, 35]. In addition to the role of BCL2 in down‐regulating the activity of ITPR, other studies report that BCL‐XL sensitizes the ITPR to low levels of inositol trisphosphate, the agonist of ITPR channels, and that BCL‐XL binds to the same region of the ITPR as BCL2 [36, 37]. The potential competition between BCL2 and BCL‐XL emphasizes the importance of the relative expression of these two proteins. Once bound to the ITPR at the mitochondrial‐associated ER membrane (MAM), BCL‐XL promotes *pro‐survival* oscillations of calcium ions, whereas BCL2 prevents *pro‐apoptotic* transients of calcium ions [38, 39].

### Calcium signaling

Proteins that were involved in calcium signaling, particularly at the MAM, showed divergence between adjuvant and neoadjuvant‐treated patients (Fig. 1B). As previously mentioned, BCL2 influences calcium signaling by inhibiting the ITPR. Due to the limits of the RPPA patient data, we could not evaluate all relevant players involved in calcium signaling, but it was possible to evaluate PERK, GRP75, and MTOR alongside BCL2 and NLRP3 (Fig. 1B). PERK, GRP75, and MTOR levels did not deviate dramatically between adjuvant‐ and neoadjuvant‐treated patients. As these proteins are cytoplasmic or plasma membrane associated, this result suggests that the relevant signaling pathway is more focused on the MAM, particularly the calcium ion regulation mitigated by ITPR channel activity.

NLRP3 is a calcium‐mediated protein associated with the inflammasome, whose activation is triggered by mitochondrial ROS production and by ER stress [40]. Once NLRP3 is activated, NLRP3 is known to trigger various types of cell death including pyroptosis, necrosis, and apoptosis [41]. Furthermore, BCL2 and BCL2‐XL suppress NLRP3 activity by binding ATP and preventing oligomerization of NLRP3 [40, 41]. Calcium activation is necessary for the NLRP3‐dependent pro‐inflammatory action to occur, which is partially mediated by the GRP75‐VDAC1‐ITPR3 complex [42, 43]. Maintenance of ER–mitochondria contacts is required for full NLRP3 activation. For instance, macrophages with reinforced MAM coupling show heightened inflammasome activity, whereas disrupting the IP3R–GRP75–VDAC1 axis attenuates the activity [44, 45, 46]. GRP75 is a glucose‐regulated protein that facilitates contact between the ER and mitochondria via the MAM and is a tether protein that links the ITPR and voltage‐dependent anion channel (VDAC1) to regulate calcium flux [47, 48]. BCL2 interferes in this pathway due to its binding to the ITPR which will inhibit the release of calcium from the ER and the transfer of calcium to the mitochondria [49, 50].

### Cellular senescence

RB1 is known to be involved in senescence, and RB1 dephosphorylation plays a role in determining a cell's apoptotic fate [28, 51]. When RB1 is inhibited (i.e., not dephosphorylated), the cell does not undergo apoptosis. Across cancers, RB1 pathway state tracks chemotherapy response whereby RB1 program loss phenocopies RB1 defects and predicts treatment sensitivity, supporting our observation that RB1 directionality may index the senescence/apoptosis balance [52]. Specifically, previous studies have indicated that BCL2 anti‐apoptotic activity prevents RB1 dephosphorylation and apoptosis thereby allowing RB1 to activate senescence‐related pathways [29]. Additionally, BCL2 has been shown to modulate Hippo signaling [53]. The Hippo signaling pathway has been shown to be involved in creating resistance to therapeutics [54] and the induction of senescence in human nucleus pulposus chondrocytes leads to an increase of the Hippo pathway [55].

Recent evidence demonstrates that the primary pathways underlying senescence are calcium signaling related [56]. Cancer treatments are known to result in cellular senescence, which occurs when the cell cycle is halted and unable to repair damage. However, in some cases, the cell never restarts division, nor does apoptosis occur, yet the cell remains in a senescent state marked by the secretion of senescent‐associated secretory phenotype (SASP) [56]. SASP production encourages an environment that is immunosuppressive and increases the proliferation of surrounding cells, which results in a recurrence of cancer [13, 56, 57, 58]. *In vivo*, it has been shown that paclitaxel accelerates cerebromicrovascular endothelial senescence and neurovascular dysfunction, which may be downstream of intracellular calcium signaling dysregulation [59]. During senescence, the interaction between ITPR and the MAM increases and there is an accumulation of calcium ions in the mitochondria [47, 60]. Due to the altered mitochondrial state, ROS production increases. The increased ROS often leads to DNA damage, which triggers DNA damage response (DDR) pathways and activates p21 to inhibit cyclin‐dependent kinases. The activation of these kinases then inhibits pro‐apoptotic protein RB1, leading to secretion of SASP proteins, such as interleukin‐6 (IL6) [61, 62, 63].

Because the RPPA panel contains limited canonical senescence readouts, we evaluated available senescence‐associated regulators (p16, p21) and SASP‐linked proteins (PAI‐1, IL‐6; RELA/NF‐κB signaling) as supportive, not definitive, markers. Only one of the documented SASP proteins, IL6, an interleukin, was available within the RPPA patient dataset. IL6 expression levels in adjuvant patients were lower after 12 weeks of chemotherapy, whereas neoadjuvant patients showed higher expression of IL6 (Fig. 1C). IL6 is a conserved SASP component across models; elevated SASP/IL6 after cytotoxic therapy has been linked to pro‐tumorigenic microenvironmental remodeling, consistent with our cohort's opposite IL6 trends by treatment timing [64]. Additional senescence‐related proteins were examined: RELA, which is an anti‐apoptotic protein involved in coordinating the DDR and SASP secretion, and p53, which is a tumor suppressor that promotes apoptosis [65, 66, 67]. P53 also inhibits Nf‐kB, which activates BCL2. Our results indicate that p53 and RELA levels were similar for neoadjuvant and adjuvant patients, possibly suggesting that p53 was unable to regulate BCL2 activity via Nf‐kB. These comparisons raise the possibility of greater engagement of senescence‐associated inflammatory/secretory signaling in the neoadjuvant group, though canonical cell cycle senescence markers available in the RPPA panel (p16/p21) did not show consistent induction, and the small cohort warrants cautious interpretation (Fig. S1).

### Long‐term implications

**Fig. 2:**
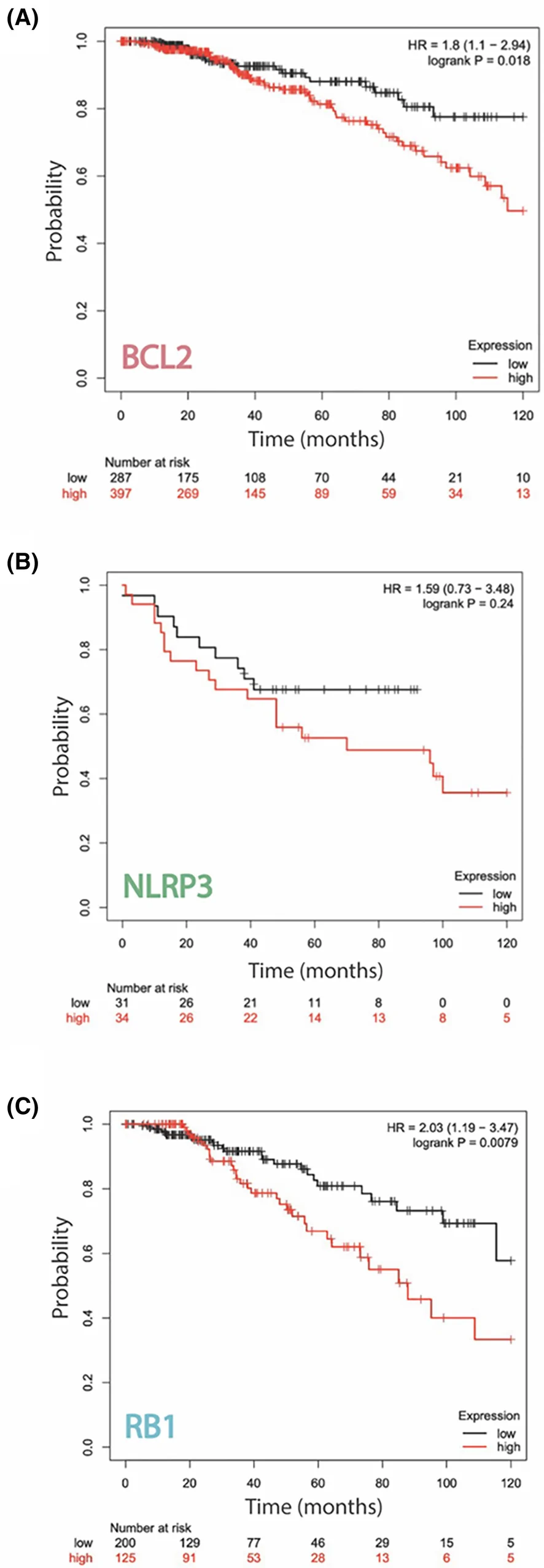
(A–C) Kaplan–Meier survival plots for proteins identified in Fig. 1. Survival plots are over the course of 120 months for BCL2 (HR = 1.8 (1.1–2.94), logrank *P* = 0.018) (A), NLRP3 (HR = 1.59 (0.73–3.48), logrank *P* = 0.24) (B), and RB1 (HR = 2.03 (1.19–3.47), logrank *P* = 0.079) (C). Survival cutoff was set to 120 months and patients were split by optimal cutoff (Red = overexpression, black = underexpression).

Our patient data was limited to the scope of the prospective study, which was the length of taxane treatment (12 weeks). However, the potential implication of these findings is better contextualized using longitudinal data. To investigate long‐term implications, Kaplan–Meier plots were generated using open‐access RPPA data. Despite low patient numbers for NLRP3, RPPA data were used instead of gene chip or mRNA data to remain consistent with the usage of proteomics data in this report. This dataset did not have stratification based on treatment setting or sampling time‐point, and they do not directly validate our findings. Rather, they serve to highlight broader clinical relevance. Because the open access data were not split by neoadjuvant and adjuvant patients, these data reflect general trends across a breast cancer cohort (*n* = 875 for BCL2 and RB1, *n* = 65 for NLRP3). The data were split by optimal cutoff based on ROC curves, and overall survival (OS) data was plotted over the course of 120 months (Fig. 2). KMplotter, a verified Kaplan–Meier curve generator was used to visualize the curves [68]. The proteins examined for the Kaplan–Meier plots were BCL2 (Fig. 2A), NLRP3 (Fig. 2B), and RB1 (Fig. 2C). For NLRP3, only data from an external study [68] was available, whereas for BCL2 and RB1, data from the TCGA RPPA was available. All three of these proteins showed the same relative pattern. For BCL2 and RB1 the high and low expression level groups were mostly in line with one another until approximately the 2.5‐year mark. After this point, patients who had high expression of BCL2 and RB1 had a lower probability of survival. In contrast, for NLRP3, the divergence occurred later. Considering that all neoadjuvant patients in the Kaplan–Meier plots exhibited BCL2 overexpression right after completing treatment, these results suggest that regulation of BCL2 levels is integral to overall survival.

Note that there is a distinction between long‐term and short‐term effects of taxane‐based treatment. Shortly after treatment, BCL2 shows an immediate increase in expression in most patients (neoadjuvant). Additionally, although the short‐term effects of NLRP3 and RB1 are less dramatic, the Kaplan–Meier plots demonstrate adverse long‐term effects associated with the overexpression of these proteins. The increase of all these proteins implicates long‐term effects in the senescent pathway, such that the buildup of senescent cells post‐taxane treatment is a result that becomes apparent months after treatment, as opposed to just weeks after treatment.

## Discussion

The purpose of this study was to investigate BCL2 and associated protein expression levels in a cohort of patients with breast cancer who underwent adjuvant or neoadjuvant taxane‐based chemotherapy. Overall, we found that breast cancer patients who received neoadjuvant chemotherapy had significantly increased levels of the protein BCL2 compared to those who received adjuvant chemotherapy. Other proteins related to BCL2, NLRP3, and RB1 also showed differences between the two groups. These differences may contribute to calcium signaling dysregulation and stress‐response states with senescence‐associated/SASP‐like features, which have been linked to therapy tolerance and recurrence. Proteomic data offers a window into the physiological state of a cell by capturing post‐translational protein level changes. Systemic proteomic monitoring has been used as an early diagnostic tool for other cancers; however, there are fewer studies that use proteomics throughout an entire treatment course [69, 70, 71]. When integrated with transcriptomic and other ‐omics tools, proteomics can help to build a more complete picture of cellular changes during chemotherapy. Though we were limited to proteomics in peripheral white blood cells, this small analysis revealed distinct patterns which can inform downstream mechanistic hypotheses. In addition, within the larger dataset, upregulation of BCL2 and related proteins was associated with a lower survival probability 2–3 years after treatment. However, this study was limited by its small size and the limited number of validated proteins available for analysis. Although Kaplan–Meier analyses of public datasets provide supportive context, these datasets lack stratification by treatment setting and cannot serve as direct validation of our pilot findings; thus, our observations should be interpreted as exploratory and hypothesis‐generating, warranting confirmation in larger, prospectively designed cohorts. The goal of cancer treatment is to both mitigate the immediate threat and prevent recurrence. Perhaps regulating the activity of BCL2 family members is one possible route to maintain long‐term cellular homeostasis and prevent recurrence of cancer metastasis. Preclinical evidence has shown that therapy‐induced senescent tumor cells can be selectively cleared by senolytics, namely ones which disrupt the interaction between BCL‐XL and BAX, demonstrating the clinical relevance of targeting BCL2‐family signaling in the management of a post‐treatment senescent response to reduce the incidence of cancer recurrence [30, 72, 73, 74]. Further research with larger sample sizes and a wider range of proteins is needed to confirm and expand upon these findings. If validated in larger datasets, we suggest that when evaluating neoadjuvant and adjuvant treatment, it is important to also consider calcium signaling dysregulation and the potential for chronic senescence.

## Conclusion

The data from our pilot, prospective study demonstrate how blood proteomics can detect molecular shifts associated with chemotherapy in a context‐dependent manner. The differences in BCL2 and its protein partners give rise to future hypotheses about how neoadjuvant vs. adjuvant treatment timing can influence systemic cellular states. Moreover, we hope to demonstrate how proteomic tools can non‐invasively track treatment‐associated stress responses, and how supplementing this approach with multi‐omic and longitudinal measurements can offer insights into diverse treatment responses and survivorship.

## Author contributions

SM analyzed data and wrote the first draft; EYI obtained and analyzed data, edited manuscript; RLR added clinical insights and edited manuscript; LMF discussed and edited manuscript; KB discussed and edited manuscript; ML obtained funding, added clinical insights, and edited manuscript; BEE conceived project, obtained funding, analyzed data, and edited manuscript. All authors approved final manuscript.

## Supporting information


**Fig. S1.** Senescence‐associated markers available in the RPPA panel.
**Table S1.** Patient demographics. Age and BMI are reported as mean ± SD.
**Table S2.** Percent change in protein expression levels from 0 to 12 weeks after taxane‐based treatment for neoadjuvant (*n* = 5) and adjuvant (*n* = 2) patients.
**Table S3.** Descriptive statistics for proteins with *P* < 0.05.
