# Supplementary material for: Blood‐based proteomic profiling reveals context‐dependent changes in BCL2‐associated signaling during taxane therapy in breast cancer patients
Source: FEBS Open Bio. 2026 Mar 26;16(9):1801–12. doi: 10.1002/2211-5463.70239 (PMC13398688; doi:10.1002/2211-5463.70239)
Supplement: Supplementary file 1 [file FEB4-16-1801-s001.docx]

**Blood-Based Proteomic Profiling Reveals Context-Dependent Changes in BCL2-Associated Signaling During Taxane Therapy in Breast Cancer Patients**

S. Munshani^1^, E.Y. Ibrahim^1*^, R.L. Rodwin^2^, L.M. Ferrucci^3^, K. Blenman^4^, M. Lustberg^4^, B.E. Ehrlich^1^

^1^Department of Pharmacology, Yale School of Medicine, New Haven, CT.

^2^Department of Pediatrics (Hematology/Oncology), Yale School of Medicine, New Haven, CT.

^3^Department of Chronic Disease Epidemiology, Yale School of Public Health, New Haven, CT.

^4^Department of Medicine (Medical Oncology), Yale School of Medicine, New Haven, CT.

**Supplementary figure and tables**

| *Age* | 47 *±*11.7 |
| --- | --- |
| *BMI* | 28.4 *±* 8.6 |
| *Stage* | 1A (n=2)  2A (n=2)  2B (n=2)  N/A (n=1) |
| *History of Smoking* | Yes (n=2)  No (n=5) |
| *Race* | White (n=5)  Black (n=1)  Hispanic (n=1) |

Supplementary Table 1: Patient demographics. Age and BMI are reported as Mean *±* SD.

| Protein | Neoadjuvant | Adjuvant |
| --- | --- | --- |
| BCL2 | +67% | –38% |
| NLPR3 | –8.0% | +68% |
| RB1 | +6.4% | +15% |
| GRP75 | +17% | –14% |
| RELA | +10% | +11% |
| P53 | +2.2% | +5.8% |
| IL6 | +11% | – 4.1% |
| PERK | +11% | +4.2% |
| MTOR | +6.5% | +6.2% |
| BAX | +14% | +26% |
| BCL-XL | +5.9% | +9.3% |
| BCLW | –4.2% | +7.3% |

Supplementary Table 2: Percent change in protein expression levels from 0 to 12 weeks after taxane-based treatment. neoadjuvant (n=5) and adjuvant (n=2) patients

|  | *Mean* | *SD* | *SEM* | *Minimum* | *25^th^ percentile* | *Median* | *75^th^ percentile* | *Maximum* |
| --- | --- | --- | --- | --- | --- | --- | --- | --- |
| *BCL2: Neoadjuvant* | 0.6602 | 0.4203 | 0.1871 | 0.1332 | 0.3040 | 0.5256 | 1.0836 | 1.1782 |
| *BCL2: Adjuvant* | -0.3789 | 0.1382 | 0.0977 | -0.4766 | -0.4766 | -0.3789 | -0.2811 | -0.2811 |
| *RB1: Neoadjuvant* | -0.0794 | 0.0982 | 0.0439 | -0.1739 | -0.1602 | -0.1242 | 0.0238 | 0.0538 |
| *RB1: Adjuvant* | 0.1546 | 0.1344 | 0.0950 | 0.0595 | 0.0595 | 0.1546 | 0.2496 | 0.2496 |
| *NLRP3: Neoadjuvant* | -0.0798 | 0.2364 | 0.1057 | -0.4932 | -0.2774 | -0.0321 | 0.0940 | 0.2174 |
| *NLRP3: Adjuvant* | 0.6781 | 0.2281 | 0.1619 | 0.5170 | 0.5170 | 0.6781 | 0.8409 | 0.8409 |

Supplementary Table 3: Descriptive statistics for proteins with p<0.05.

Supplementary Figure 1: Senescence-associated markers available in the RPPA panel
